# Assessing the Chemical Composition and Antimicrobial Activity of Essential Oils from Brazilian Plants—*Eremanthus erythropappus* (Asteraceae), *Plectrantuns barbatus*, and *P. amboinicus* (Lamiaceae)

**DOI:** 10.3390/molecules20058440

**Published:** 2015-05-11

**Authors:** Nara O. dos Santos, Bruna Mariane, João Henrique G. Lago, Patricia Sartorelli, Welton Rosa, Marisi G. Soares, Adalberto M. da Silva, Harri Lorenzi, Marcelo A. Vallim, Renata C. Pascon

**Affiliations:** 1 Instituto de Ciências Ambientais, Químicas e Farmacêuticas, Universidade Federal de São Paulo, 09972-270 Diadema, SP, Brazil; E-Mails: nara.oshiro@gmail.com (N.O.S.); bruna_mariane@yahoo.com.br (B.M.); joao.lago@unifesp.br (J.H.G.L.); patty.sart@unifesp.br (P.S.); marcelo.vallim@gmail.com (M.A.V.); 2 Instituto de Química, Universidade Federal de Alfenas, 37130-000 Alfenas, MG, Brazil; E-Mails: welton_rosa@hotmail.com (W.R.); marisigs@gmail.com (M.G.S.); 3 Departamento de Química, Universidade Federal de Viçosa, 36570-000 Viçosa, MG, Brazil; E-Mail: adalbert31@hotmail.com; 4 Instituto Plantarum de Estudos da Flora. Av. Brasil, 2000, 13460-000 Nova Odessa, SP, Brazil; E-Mail: hlorenzi@plantarum.com.br

**Keywords:** *Eremanthus erythropappus*, *Plectranthus barbatus*, *Plectranthus amboinicus*, essential oils, chemical composition, antimicrobial activity

## Abstract

The chemical composition and antimicrobial activity of essential oils obtained from three Brazilian plant species—leaves and branches of *Eremanthus erythropappus* (Asteraceae), leaves of *Plectranthus barbatus*, and leaves of *P. amboinicus* (Lamiaceae)—were determined. Analysis by GC/MS and determination of Kovats indexes both indicated δ-elemene (leaves—42.61% and branches—23.41%) as well as (−)-α-bisabolol (leaves—24.80% and stem bark—66.16%) as major constituents of *E. erythropappus* essential oils. The main components of leaves of *P. barbatus* were identified as (*Z*)-caryophyllene (17.98%), germacrene D (17.35%), and viridiflorol (14.13%); whereas those of leaves of *P. amboinicus* were characterized as *p*-cymene (12.01%), γ-terpinene (14.74%), carvacrol (37.70%), and (*Z*)-caryophyllene (14.07%). The antimicrobial activity against yeasts and bacteria was assessed in broth microdilution assays to determine the minimum inhibitory concentration (MIC) necessary to inhibit microbial growth. In addition, the crude oil of branches of *E. erythropappus* was subjected to chromatographic separation procedures to afford purified (−)-α-bisabolol. This compound displayed biological activity against pathogenic yeasts, thus suggesting that the antimicrobial effect observed with crude oils of *E. erythropappus* leaves and branches may be related to the occurrence of (−)-α-bisabolol as their main component. Our results showed that crude oils of Brazilian plants, specifically *E. erythropappus*, *P. barbatus*, and *P. amboinicus* and its components, could be used as a tool for the developing novel and more efficacious antimicrobial agents.

## 1. Introduction

Essential oils are aromatic liquids frequently obtained by steam distillation of several plant materials [1] and commonly composed of volatile metabolites such as terpenoids and phenylpropanoids [2,3]. Several reports describe the use of crude essential oils and their components in folk medicine, indicating the pharmaceutical potential of these volatile compounds [4]. Additionally, an important application of essential oils stems from their potential antimicrobial activity against bacteria, fungi and yeasts [5]. The antibacterial properties of essential oils and their components are exploited in different commercial products, such as root canal sealers [6] and antiseptics [7]. 

*Eremanthus erythropappus* (DC) McLeisch (Asteraceae) is commonly known as “candeia” and has been used in traditional medicine as an antimicrobial agent [8]. Previous studies reported on the characterization of mono- and sesquiterpenes in essential oils extracted from leaves, branches and bark [9]. Furthermore, both the essential oil extracted from this plant and the sesquiterpene β-bisabolene showed the potential to restore the effectiveness of ampicillin against resistant *Staphylococcus aureus* [10]. *Plectranthus barbatus* Andrews (Lamiaceae) is popularly known as “falso boldo” and has been used in traditional medicine for treating heart and central nervous diseases as well as digestive and respiratory disorders [11]. Previous studies described the occurrence of hydrocarbon sesquiterpenes in the essential oil extracted from leaves [12,13]. In addition, belonging to the *Plectranthus* genus, *P. amboinicus* (Lour.) Spreng has been used in the Brazilian folk medicine to treat skin diseases, with its leaves being used topically in the treatment of furuncles and superficial mycoses, as well as in the treatment of digestive diseases [14]. As described, the essential oil of their leaves is composed of mono- and sesquiterpenoids [15].

As part of our continuous research aiming at the discovery of new antimicrobial agents from Brazilian plant species [16,17,18,19,20], in this work we determined the chemical composition of essential oils extracted from three different selected plant species: *E. erythropappus* (leaves and branches), *P. barbatus* (leaves), and *P. amboinicus* (leaves). Additionally, their antimicrobial potential against yeasts and bacteria (including some strains that displayed resistance to antibiotics [21,22] was assessed in broth microdilution assays and the minimum inhibitory concentration (MIC) was thus determined. 

## 2. Results and Discussion

### 2.1. Chemical Composition of the Essential Oils Obtained from E. erythropappus, P. barbatus and P. amboinicus

Individual hydrodistillation of the fresh leaves and branches of *E. erythropappus* afforded yellow viscous oils (yields w/w—leaves 0.12% and branches 0.43%, respectively), while that of leaves of *P. barbatus* and *P. amboinicus* provided colorless oils (yields w/w—0.45% and 0.12%, respectively). The crude essential oils were analyzed by FID-GC (RtX-5 capillary column) and GC-MS. Individual compounds were assigned according to their Kovats indexes in conjunction with a comparison of the experimentally obtained mass spectra to those described in a mass spectra library (NIST 107) and in the literature [23]. The chemical composition of the oils studied and the relative quantity of each compound identified therein are shown in Table 1.

In total, 14 compounds were identified in the oils of leaves and branches of *E. erythropappus*, which altogether accounted for 98.78 and 99.93% of the total oil composition, respectively. Similar compositions were observed for both oils, with sesquiterpenes being their major constituents (leaves: 88.72%; branches: 91.86%). Among the identified compounds, δ-elemene and (−)-α-bisabolol were the main derivatives, corresponding to 42.61% (leaves)/23.41% (branches) and 24.80% (leaves)/66.16% (branches), respectively. However, the oil of the leaves also contained high amounts of (*Z*)-caryophyllene and germacrene D (10.01 and 10.45%, respectively), while traces of these sesquiterpenes were detected in in the oil obtained from branches. Monoterpenes were detected in smaller concentrations in both of the oils analyzed, with α- and β-pinenes being the main compounds. In addition, one phenylpropanoid (safrol) was found to be present in the oil of leaves (0.41%), albeit at a lower concentration when compared to the oil extracted from branches (2.14%). Comparatively, the oils obtained from fresh leaves of *E. erythropappus* collected in the city of Juiz de Fora, Minas Gerais State, Brazil [9], displayed a different profile than that detected in the present study since it consisted mainly of (*E*)-caryophyllene (21.8%), germacrene D (14.9%), and α-copaene (10.2%). Similarly, when compared to our results, the oxygenated sesquiterpene α-bisabolol was detected in a smaller quantity (2.2%) in the oil from leaves, while it was observed to be predominant in branches (91.3%).

The essential oils of *P. barbatus* and *P. amboinicus* were composed of 34 identified compounds (Table 1), which altogether accounted for 91.08% and 97.35% of the total oil composition, respectively. In the essential oil of *P. barbatus*, sesquiterpenes were the major constituents (81.25%), followed by monoterpenes in lower concentrations (9.83%); whereas the main compounds found in the essential oil of *P. amboinicus* were monoterpenes (70.11%), followed by sesquiterpenes (27.24%). With regard to *P. barbatus* oil, its major chemical compounds were (*Z*)-caryophyllene (17.98%), germacrene D (17.35%), besides viridiflorene (14.13%), cyclosativene (9.94%), and α-pinene (8.85%). In a previous study [13], germacrene D (24.86%) and farnesene (21.40%) were both described as the main constituents of the essential oil of leaves of *P. barbatus* from Brazil. Additionally, in the essential oil obtained from a species grown in Portugal, the main constituent was α-pinene (67.00%), which was detected during this study but at a smaller concentration (8.85%) [24]. The main compounds identified in the oil of leaves of *P. amboinicus* were carvacrol (37.70%), γ-terpinene (14.74%), (*Z*)-caryophyllene (14.07%), and *p*-cymene (12.01%). Carvacrol had been previously described in the essential oil of this species in different studies [15,25,26,27] in concentrations ranging from 28% to 70%. In another study [12], the sesquiterpenes (Z)-caryophyllene (25.53%) and caryophyllene oxide (9.76%) were shown to be predominant in the essential oil of leaves, while no monoterpenes were detected, therefore constituting a different profile that those of all oils of *P. amboinicus* previously studied. 

**Table 1:** Chemical composition of essential oils obtained from *Eremanthus erythropappus* (EE), *Plectranthus barbatus* (PB), and *Plectranthus amboinicus* (PA).

Compound ^a^	KI	*Relative amount (%)*			
		EE leaves	EE branches	PB leaves	PA leaves
tetrahydrocitronellene	936	-	-	0.98	-
α-pinene	939	3.31	2.01	8.85	0.24
camphene	953	0.37	0.15	-	-
β-pinene	980	4.21	2.81	-	-
β-myrcene	991	0.33	0.13	-	0.97
α-phellandrene	1002	-	-	-	0.12
α-terpinene	1007	-	-	-	1.96
*p*-cymene	1024	-	-	-	12.01
limonene	1031	1.43	0.83	-	0.46
γ-terpinene	1059	-	-	-	14.74
terpin-4-ol	1177	-	-	-	1.39
safrole	1285	0.41	2.14	-	-
thymol	1290	-	-	-	0.52
carvacrol	1298	-	-	-	37.7
δ-elemene	1339	42.61	23.41	-	-
α-cubebene	1351	-	-	0.45	-
cyclosativene	1368	-	-	9.94	-
α-copaene	1376	-	-	0.97	-
β-bourbonene	1384	-	-	1.68	-
β-cubebene	1390	-	-	0.89	-
β-longipinene	1398	-	-	0.88	-
(*Z*)-caryophyllene	1404	10.01	0.11	17.98	14.07
β-cedrene	1418	-	-	5.68	-
*trans*-α-bergamotene	1434	-	-	-	8.19
β-humulene	1440	-	-	0.32	-
α-humulene	1454	-	0.13	1.13	3.83
(*E*)-β-farnesene	1456	-	-	-	0.39
γ-muurolene	1477	-	-	0.53	-
germacrene-D	1480	10.45	0.16	17.35	-
β-selinene	1485	-	-	2.13	-
viridiflorene	1493	-	-	14.13	-
germacrene A	1503	0.22	0.16	0.81	-
7-*epi*-α-selinene	1517	-	-	1.67	-
δ-cadinene	1524	-	-	1.40	-
germacrene B	1556	0.31	0.18	0.25	-
nerolidol	1564	0.32	1.55	-	-
caryophyllene oxide	1581	-	-	2.80	-
1-*epi*-cubenol	1627	-	-	0.26	-
*(E)*-*epi*-14-hydroxy-9-caryophyllene	1669	-	-	0.52	-
(−)-α-bisabolol ^b^	1683	24.80	66.16	-	-
Monoterpenes		9.65	5.93	9.83	70.11
Sesquiterpenes		88.72	91.86	81.25	27.24
Phenylpropanoids		0.41	2.14	-	-
TOTAL		98.78	99.93	91.08	97.35

^a^ Individual compounds were assigned according to their Kovats indexes in conjunction with a comparison of the experimentally obtained mass spectra to those described in a mass spectra library (NIST 107) and in the literature [23]. ^b^ The absolute configuration was determined by optical rotation (see the Experimental section).

### 2.2. Antimicrobial Activity—Disk Diffusion Assay

The effects on microbial growth of essential oils of leaves and branches of *E. erythropappus*, as well as leaves of *P. barbatus* and *P. amboinicus*, were primarily evaluated in a disk diffusion assay. Table 3 describes all the yeast and bacterial strains tested with this qualitative method and the obtained results. These data show that seven microorganisms (*S. epidermidis*; *C. albicans*; *C. neoformans* serotype A; *C. gattii* serotype B; *C. gattii* serotype C; *C. neoformans* serotype D; and *S. cerevisiae*) were sensitive to at least one compound, thus producing a visible growth inhibition halo, whereas the growth of the remaining strains was not inhibited and, for this reason, not further explored. Those seven microbial strains were thus investigated in Minimal Inhibitory Concentration (MIC) assays in order to quantitatively determine the quantity of oil/compound necessary to inhibit microorganism growth.

### 2.3. Antimicrobial Activity—MIC Testing

As seen in Table 2, the obtained results allow us to conclude that the essential oils of *P. amboinicus* were able to inhibit the growth of *S. epidermidis*, *C. albicans*, *C. gattii* (B), *C. gattii* (C), *C. neoformans* (D), and *S. cerevisiae*. However, the quantity of essential oil necessary to inhibit the microbial growth was higher when compared to chloramphenicol and fluconazole [*S. epidermidis* (7×); *C. albicans* (3×); *C. gattii* (B) (0.2×); *C. gattii* (C) (5×); *C. neoformans* (D) (0.67×); and *S. cerevisae* (0.53×)]. 

*C. neoformans* (serotype A) growth was not inhibited at any concentration of the crude essential oil of *P. amboinicus*, whereas the growth inhibition observed for *C. gattii* (C) was only 50% ± 18% when the concentration of essential oil was five times as high as that of fluconazole. On the other hand, the other serotypes tested (*C. gattii—*serotype B and *C. neoformans—*serotype D) exhibited growth inhibition rates above 80% with a similar quantity of fluconazole. Noteworthy is the fact that the essential oil of *P. amboinicus*, among all oils tested, was the only one observed to inhibit the growth of *S. epidermidis* and *S. cerevisiae*. Essential oils obtained from plants of the genus *Plectranthus* displayed antibacterial and antifungal activities as previously reported in the literature [28]. Our results are consistent with this observation, especially with regard to the genus *Candida*, whose growth has been reported as being affected by essential oils obtained from several species [14,28,29,30]. To our knowledge, this is the first report on the biological activity of *Plectranthus* genus against *Cryptococcus* spp., which could be considered as an important finding since these fungi can cause fatal invasive infections, especially in immunocompromised patients [18]. Furthermore, the oil of *P. barbatus* was effective against two *C. neoformans* serotypes, namely A and C, at a concentration of 59.0 µg/mL, inhibiting 73% ± 15% and 82% ± 11% of their growth, respectively. 

Similarly, the essential oils of leaves and branches from *E. erythropappus* showed activity against the pathogenic yeasts tested. When compared to fluconazole, the quantity of essential oil of stem bark necessary to produce an 81% ± 10% growth inhibition was 18-fold lower, whereas a quantity of essential oil of leaves that is 147-fold lower than that of the positive control causes 88% ± 4% growth inhibition in *C. albicans*. Aiming to associate the presence of α-bisabolol, the main compound in both crude oils, with their antimicrobial activity, this compound was purified by using column chromatography. As observed in Table 2, purified (−)-α-bisabolol as well as the crude essential oils did inhibit the growth of *C. albicans* to different degrees. The sesquiterpene (−)-α-bisabolol was shown to cause a 43% ± 10% growth inhibition at a concentration 24-fold lower than that of fluconazole. Furthermore, the essential oil of leaves showed no activity against *C. neoformans* serotype A, while the essential oil of branches produced a 67% ± 2% growth inhibition of this yeast. After purification, (−)-α-bisabolol (with a quantity 12.5-fold lower than that of fluconazole) was able to cause an 85% ± 4% growth inhibition of *C. neoformans* serotype A. In turn, the growth of *C. gatti* serotype B was inhibited by the essential oil of branches (86% ± 7%) and leaves (100% ± 1%) with quantities 4.6- and 35-fold lower than that of fluconazole, respectively. Consequently, only (−)-α-bisabolol and the essential oil obtained from the branches of *E. erythropappus* were able to inhibit the growth of *C. gatti* serotype C (98% ± 5% and 81% ± 5%, respectively) at concentrations that were 5.7-fold and 1.1-fold lower than that of fluconazole, respectively. *C. neoformans* serotype D had its growth inhibited by (−)-α-bisabolol (92% ± 5%) and the essential oil of branches (94% ± 14%), with these effects being observed with quantities approximately 23- and 2.2-fold lower than that of fluconazole. Therefore, the results obtained suggest that (−)-α-bisabolol could be responsible, at least in part, for the detected antimicrobial activity of essential oils of leaves and branches of *E. erythropappus*. 

The antimicrobial potential against *Candida albicans* had been previously described for α-bisabolol isolated from *Laserpitium zernyi* and *Eryngium tricuspidatum* [31,32]. In both cases, α-bisabolol was detected as the main constituent (30.9% and 32.6%). In accordance with these results, ours too indicated a high activity for this sesquiterpene (at 1.04 µg/mL) as an agent against *Candida albicans*. 

In regard to *Cryptococcus*, this is the first report on (−)-α-bisabolol acting as an agent against the main serotypes that can cause disease in humans and animals. As shown in Table 2, a remarkable anticryptococcal activity was observed, which could lead to novel research and treatment possibilities. Overall, oils of *E. erythropappus*, *P. barbatus*, and *P. amboinicus* could be an alternative when fighting microbes that are resistant to the canonical antimicrobial therapies available such as fluconazole and amphotericin B. 

**Table 2:** Minimum inhibitory concentrations (MICs) in µg/mL obtained from broth microdilution assays with the essential oils of *Eremanthus erythropappus*, *Plectranthus barbatus*,and *P. amboinicus*.

Species	*Eremanthus erythropappus*			*Plectranthus barbatus*leaves	*Plectranthus amboinicus*Leaves	Positive Control
	leaves	branches	(−)-α-bisabolol			
*S. epidermidis*	NI	NI	NI	NI	31.0 (98% ± 2%)	40.0 ^b^
*C. albicans*	0.17 (88% ± 4% ^a^)	1.35 (81% ± 10%)	1.04 (43% ± 10%)	NI	80.0 (91% ± 11%)	25.0 ^c^
*C. neoformans* (A)	NI	10.80 (67% ± 2%)	1.04 (85% ± 4%)	59.0 (73% ± 15%)	NI	13.0 ^c^
*C. gattii* (B)	0.71 (100% ± 1%)	5.40 (86% ± 7%)	NI	NI	30.0 (85% ± 6%)	25.0 ^c^
*C. gattii* (C)	NI	5.40 (81% ± 17%)	1.04 (98% ± 5%)	59.0 (82% ± 11%)	30.0 (50% ± 18%)	6.0 ^c^
*C. neoformans* (D)	NI	2.70 (94% ± 14%)	0.26 (92% ± 5%)	NI	10.0 (87% ± 5%)	6.0 ^c^
*S. cerevisae*	NI	NI	NI	NI	20.0 (97% ± 6%)	13.0 ^c^

^a^ Numbers in parentheses represent the mean percentage inhibition at each MIC, ± standard deviation; ^b^ chloramphenicol; ^c^ fluconazole; NI: no inhibition.

## 3. Experimental Section

### 3.1. General Experimental Procedures

Silica gel 60 and silica gel 60 PF_254_ (Merck, New Jersey, NJ, USA) were used for column and TLC separations, respectively, whereas all solvents used were of analytical grade and purchased from CAAL (São Paulo, Brazil). Linear *n*-alkane (C_8_–C_20_) reference standards, as well as all culture media and standard antibiotic discs of fluconazole and chloramphenicol, were obtained from Sigma-Aldrich Chemical Co. (St. Louis, MO, USA). All other chemicals were purchased from Merck (Darmstadt, Germany). ^1^H (300 MHz) and ^13^C (75 MHz) NMR spectra were recorded on a Bruker spectrometer (UltraShield 300 Avance III spectrometer) using CDCl_3_ as solvent and TMS as an internal standard, both purchased from Sigma-Aldrich (St. Louis, MO, USA). Optical rotation measurements were performed on a JASCO DIP-370 digital polarimeter (Na filter, λ = 588 nm) using CHCl_3_ as solvent.

### 3.2. Plant Material

Leaves and branches of *Eremanthus erythropappus* (DC) McLeisch (Asteraceae) were collected randomly from one individual tree in Pouso Alegre and Caeté, cities located in Minas Gerais State, Brazil, in December 2011. Voucher specimens were compared with those deposited at the herbarium of *Instituto de Botânica* (São Paulo, Brazil). Leaves of *Plectranthus barbatus* Andrews and *P. amboinicus* (Lour.) Spreng. (Lamiaceae) were collected from different trees in March 2014, in Nova Odessa, a city in São Paulo State, Brazil. Voucher specimens of *Plectranthus barbatus* and *P. amboinicus* were deposited in the *Herbarium Plantarum* (São Paulo, Brazil).

### 3.3. Hydrodistillation of the Essential Oils

Fresh leaves and branches of *Eremanthus erythropappus,* as well as leaves of *P. barbatus* and *P. amboinicus*, were individually hydrodistilled for four hours in a Clevenger type apparatus [33]. The essential oils were extracted from the aqueous fraction using CH_2_Cl_2_ (3 × 5 mL). The combined organic fractions were subsequently dried over anhydrous Na_2_SO_4_, after which the solvent was evaporated and the oil was finally stored at 4 °C in the absence of light.

### 3.4. Gas Chromatography Analysis (GC)

The crude essential oils were analyzed by GC, using a Shimadzu GC-2010 gas chromatograph, equipped with an FID-detector and an automatic injector (Shimadzu AOC-20i, Kyoto, Japan). As the stationary phase, an RtX-5 capillary column (5% phenyl, 95% polydimethylsiloxane, 30 m × 0.32 mm × 0.25 μm film thickness; Restek, Bellefonte, PA, USA) was used with helium as the carrier gas (flow rate: 1 mL/min). The oven temperature was raised from 60 °C to 280 °C at a rate of 3 °C/min and subsequently kept at 280 °C for another ten minutes. The injector temperature was 220 °C and the detector (FID) was kept at 280 °C. Composition percentages were obtained from electronic integration of the FID output and a series of linear *n*-alkanes (C_8_–C_20_), which were used as reference points for the determination of the Kovats indexes (KI).

### 3.5. Gas Chromatography—Mass Spectrometry (GC-MS) Analysis 

GC-MS analysis was carried out using a Shimadzu GC-17A chromatograph connected to an MS-QP-5050A mass spectrometer. The GC analysis was carried out with an RtX-5 capillary column (5% phenyl, 95% polydimethylsiloxane, 30 m × 0.32 mm × 0.25 μm film thickness; Restek, Bellefonte, PA, USA) and the operating conditions were identical with those described in the previous section. Retention indices for all compounds were determined according to the Kovats indices (KI), as described in the previous section. The EI-MS analysis was carried out under an ionization voltage of 70 eV and an ion source temperature of 230 °C. The identification of individual compounds was achieved by comparing the KI values recorded, in conjunction with the matching mass spectrometric fragmentation patterns, with those of a mass spectra library (NIST 107), published MS fragmentation patterns [23], and/or MS spectra of authentic compounds.

### 3.6. Main Component Isolation from the Essential Oil of Branches of Eremanthus erythropappus 

The essential oil of branches of *E. erythropappus* (800 mg) was subjected to column chromatography on silica gel, eluted with CH_2_Cl_2_ (100 mL) and CH_2_Cl_2_/MeOH mixtures of increasing polarities [99:1 (100 mL); 98:2 (50 mL); and 9:1 (50 mL)] to afford 45 fractions. These fractions were analyzed by TLC as well as FID-GC, and those showing similar compositions were pooled into four groups (I–IV). Group II (520 mg) was composed by pure α-bisabolol (99.7%). After determining their optical specific rotation and comparing the data obtained with those described in the literature [34], the sesquiterpene (−)-α-bisabolol was identified.

*(−)-α-Bisabolol.* Colorless viscous oil. [α]^25^_*D*_ −49.0 (*c* 0.1, CHCl_3_). EI-MS *m/z* (rel. int.): 222 (8), 204 (32), 161 (10), 119 (62), 109 (84), 93 (38), 69 (98), 43 (100), 41 (89). ^1^H-NMR (CDCl_3_) δ/ppm: 5.38 (H-5, m), 5.12 (H-7, m), 1.68 (H-12, s), 1.64 (H-13, s), 1.71 (H-14, s), 1.18 (H-15, s). ^13^C-NMR (CDCl_3_) δ/ppm: 42.7 (C-1), 26.7 (C-2), 30.8 (C-3), 133.7 (C-4), 120.4 (C-5), 22.9 (C-6), 124.5 (C-7), 21.9 (C-8), 39.9 (C-9), 74.0 (C-10), 131.1 (C-11), 25.5 (C-12), 17.4 (C-13), 23.1 (C-14), 25.5 (C-15).

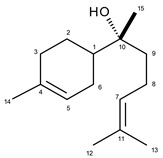


### 3.7. Media, Antibiotics, and Growth Conditions

Yeasts were cultivated on agar plates containing YPD (1% yeast extract, 2% peptone, 2% dextrose, and 2% agar) or RPMI1640 (Sigma). Gram-negative bacteria were grown in LB (0.5% yeast extract, 1% tryptone, 1% NaCl, and 2% agar), and Gram-positive bacteria were tested in BHI (Himedia). Fluconazole (Sigma) was used as the positive control for yeasts, and chloramphenicol (Sigma) was the positive control for bacteria. Essential oils were diluted in DMSO or saline (0.9%) plus Tween 80 (0.5%) and then spotted on 5 mm sterile filter paper [18].

### 3.8. Microorganism Strains

In this study, the crude essential oils obtained from branches and leaves of *E. erythropappus*, from leaves of *P. barbatus* and *P. amboinicus*, as well as the sesquiterpene (−)-α-bisabolol were all evaluated against six bacterial and 12 yeast species, as described in the Table 3. 

**Table 3:** Target strains used in antimicrobial activity assays.

Species	Designation
Yeasts	
*Candida dubliniensis*	ATCC 7978
*Candida tropicalis*	ATCC 13803
*Candida albicans*	ATCC 18804
*Candida glabrata*	ATCC 90030
*Candida parapsilosis*	Clinical isolate 68
*Candida krusei*	Clinical isolate 9602
*Candida albicans*	CBMAI 560
*Cryptococcus grubii*	KN99α (serotype A)
*Cryptococcus gattii*	NIH312 (serotype C)
*Cryptococcus gattii*	R265 (serotype B)
*Cryptococcus neoformans*	JEC21 (serotype D)
*Saccharomyces cerevisiae*	BY4742
Bacteria	
*Escherichia coli*	-
*Serratia marcescens*	CBMAI 469
*Pseudomonas aeruginosa*	CBMAI 602
*Streptococcus equi*	CBMAI 264
*Staphylococcus epidermidis*	CBMAI 604
*Enterococcus fecalis*	-

### 3.9. Disk Diffusion Assay 

Antimicrobial activity was evaluated by the disk diffusion method according to the Clinical and Laboratory Standards Institute (CLSI, OPAS M2-A8) with modifications [18]. Thin agar plates were prepared with 10 mL of YPD (yeast), LB (Gram-negative) and BHI (Gram-positive) media. Three milliliters of liquid cultures were grown at 30 °C with aeration (150 rpm) overnight on YPD (yeast), LB (Gram-negative) or BHI (Gram-positive). Top agar was prepared by mixing 100 µL of each culture with 10 mL of soft agar medium for confluent plates (YPD, LB or BHI plus 1% agar) and poured on top of the thin agar layer (2% agar medium). Sterilized 5-mm filter paper disks were then impregnated with 20 µL of essential oils or (−)-α-bisabolol diluted in DMSO. The disks were then placed on top of agar plates and incubated at 30 °C for 24 or 48 h, depending on the microorganism. Hygromycin (1 mg) and chloramphenicol (200 µg) were used as positive controls for yeasts and bacteria, respectively. Negative controls were prepared by impregnating paper disks with the same amount of DMSO used to dilute the essential oils. All tests were performed in triplicate. The inhibition zone (IZ) was determined by measuring the whole halo diameter (mm) and dividing it by the disk size (5 mm). 

### 3.10. Minimum Inhibitory Concentration (MIC)

Microdilution tests were conducted according to the Clinical and Laboratory Standards Institute (CLSI) guidelines: OPAS1 M27-A2 for yeasts, and OPAS M7-A6 for bacteria, with modifications; using sterile 96 well microtiter plates in a total volume of 100 µL/well. Microorganism cultures were grown in 3 mL medium (RPMI 1640 for yeasts, and BHI for bacteria) in test tubes overnight at 30 °C in a rotary shaker (150 rpm). The cultures were then diluted and adjusted to the final concentration of 1–2 × 10^2^ CFU/well (yeasts) and 5 × 10^4^ CFU/well (bacteria). Viability and count tests were performed on YPD and BHI plates (100 µL of cells). Two-fold serial dilutions of the essential oils, (−)-α-bisabolol, and reference standards were tested. Sterilization controls containing solely medium, and growth controls containing cells and DMSO (10 µL) or saline (10 µL) and Tween 80 were included as negative and positive controls, respectively. Depending on the microorganism to be grown, the microtiter plates were incubated at 30 °C for 24 or 48 h. Microorganism growth was determined by reading the absorbance at 530 nm in a plate reader (Logen, MT-960), and the minimum inhibitory concentration was considered as the lowest concentration at which at least 80% of growth was inhibited. All tests were performed in triplicate. The concentration range for each agent was as follows: (−)-α-bisabolol: 1.04–0.016 µg/mL; Essential oils: 80.0–0.01 µg/mL; fluconazole: 50.00–0.78 µg/mL; and chloramphenicol: 40.00–3.01 µg/mL.
